# Recipe for Hydrogels With Tunable Relaxation and Diffusion Properties for Use as MRI Test Materials

**DOI:** 10.1002/mrm.70120

**Published:** 2025-10-05

**Authors:** V. Fritz, F. Schick

**Affiliations:** ^1^ Section on Experimental Radiology, Department of Diagnostic and Interventional Radiology University of Tuebingen Germany

**Keywords:** DWI, MRI, phantoms, quantitative, relaxometry

## Abstract

**Purpose:**

To develop and evaluate a preparation protocol (recipe) for hydrogels with specific relaxation times and ADC values to be used as tissue‐like test materials for MRI experiments at 3 Tesla.

**Methods:**

Gd‐DTPA, agarose, and soy lecithin were used as modulators for T1, T2, and ADC. First, systematic measurements were performed to determine the relaxation‐ and diffusion‐modifying properties of the single substances and combinations of them. An algorithm was developed to determine the necessary concentrations of the ingredients to achieve predetermined sets of target values (T1, T2, and ADC). To validate this approach, hydrogels mimicking the relaxation and diffusion properties of different tissues (pancreas, white matter, fibroglandular tissue, liver, prostate) were prepared and evaluated. All measurements (relaxometry, diffusion‐weighted imaging) were performed on a 3 Tesla clinical scanner at 20°C.

**Results:**

The proposed method allowed the preparation of hydrogels with specific diffusion and relaxation properties by adjusting the concentrations of Gd‐DTPA, agarose, and soy lecithin. Test phantoms containing hydrogels for simulation of various tissue types showed good agreement between targeted and measured properties, with deviations of less than 8% for T1, 7.5% for T2, and 11.5% for ADC. With the present approach, the properties (T1, T2, and ADC) of most known tissue classes could be well approximated; only the gray matter of the brain was slightly outside the selectable range. Temporal stability over 3 months was acceptable.

**Conclusion:**

This work provides a relatively simple, inexpensive, and reproducible method for the preparation of hydrogels with independently adjustable T1, T2, and ADC values at 3 T.

## 1 Introduction

Suitable tissue‐mimicking materials in so‐called tissue phantoms are becoming increasingly important for testing new sequences and especially in quantitative magnetic resonance imaging (MRI) [1, 2, 3, 4]. While in vivo studies with human subjects are essential for clinical translation, early‐stage validation and proof of concept are often more efficiently conducted using MRI phantoms.

Phantom materials typically consist of liquids or gels with well‐defined and stable MRI properties, including relaxation times (T1, T2) or the apparent diffusion coefficient (ADC) [4]. Relaxation and diffusion properties can be modulated by adding paramagnetic substances (e.g., Gd‐DTPA, CuSO4), polymers (e.g., PVP, PEG), or by adjusting the concentration of gelling agents (e.g., agarose, carrageenan) [5, 6, 7, 8, 9, 10, 11, 12, 13, 14]. Despite the wide range of available phantom designs, a fundamental limitation remains: most existing materials in phantoms were designed to mimic only a single MRI parameter (e.g., T1, T2, or ADC), while leaving the others uncontrolled, which limits their ability to replicate the complex signal characteristics seen in biological tissues [1, 15].

In vivo, tissues exhibit characteristic combinations of multiple MRI parameters, inherently linked to their structural and biochemical composition [16, 17]. For instance, healthy liver tissue at 3 Tesla is characterized by a T1 relaxation time of 800–1000 ms, a T2 relaxation time of 35–50 ms, and an ADC of ˜1 ⋅ 10^−3^ mm^2^/s [18, 19, 20]. In addition, factors such as tissue perfusion, magnetization transfer, fat and iron content might play a crucial role [21]. Each of these parameters contributes to the acquired MR signal, meaning that quantitative MRI methods designed to quantify a specific parameter (e.g., T2) may be affected by others (e.g., T1 or diffusion) [22].

Very few approaches have been reported allowing the simultaneous and independent adjustment of multiple MRI parameters within a single test material. Most of these focus on relaxometry‐based tuning of T1 and T2 or on simultaneous modulation of fat fraction and R2* (via iron content) [8, 15, 23, 24, 25]. However, as noted by Keenan et al. [1], it has not yet been possible to create a material that simultaneously represents a specific T1, a specific T2, and a specific ADC value in the same voxel.

A major challenge in multi‐parametric phantom development is the interdependence of parameter‐modifying agents [4]. The preparation of a hydrogel with specific T1, T2, and ADC values requires at least three different components with different chemical properties: a T1 modifier, a T2 modifier, and an ADC modifier. However, a substance intended to adjust one MR property often influences others, making independent parameter control challenging. For example, agents used to modify the diffusion properties usually also affect the relaxation times of materials used in phantoms [9, 26, 27]. Similarly, relaxation modifiers do not act exclusively on T1 or T2 but influence both [8]. In addition, ensuring compatibility between the selected components is critical—that is, there is little or no interaction between them and their individual effects remain intact in the presence of the other substances. Undesired interactions, as seen between GdCl_3_ and agarose, lead to unexpected shifts in target values [4, 28]. In summary, a suitable triple must be chemically compatible while allowing independent adjustment of a wide range of relaxation times and ADC values.

In this context, Gd‐DTPA, agarose, and soy lecithin were found to be promising candidates. The combination of Gd‐DTPA and agarose has already been established as a suitable pair for independent adjustment of T1 and T2 [4]. In addition, soy lecithin has recently been identified as a promising ADC modifier that, when combined with agarose, allows for largely independent control of ADC and T2 [27]. This raises the question of whether these three substances (Gd‐DTPA, agarose, and soy lecithin) remain effective when combined into a single mixture and whether they allow simultaneous and independent adjustment of T1, T2, and ADC—the motivation of this study. In this work, the production of hydrogels with specific diffusion and relaxation properties by adjusting the concentrations of Gd‐DTPA, agarose, and soy lecithin will therefore be presented. The work is divided into three parts: First, the relaxation and diffusion‐modifying properties of the single substances, as well as their compatibility in combination, were systematically investigated. Second, based on the resulting findings, a recipe for the production of a hydrogel with a desired set of properties (T1, T2, and ADC) was developed and used to build test phantoms with hydrogels mimicking the diffusion and relaxation properties of different tissues. Finally, the achieved values (T1, T2, and ADC) of the produced hydrogels were measured and compared with the intended target values. Deviations were systematically determined and the target range achievable with the selected approach was identified. In addition, the reproducibility of the preparation process and the temporal stability of the gels were tested.

## 2 Methods

### 2.1 Selection of Materials

Aqueous solutions were prepared in order to be able to reproduce lean tissue (without significant fat content). Known ingredients were chosen that can be mixed together and which produce hardly any additional spectral components in the ^1^H spectrum. The following materials were used:

- Base solvent: Deionized water (Carl Roth, Karlsruhe, Germany) was used as the basis for the hydrogels.
- Gadopentetate dimeglumine (Gd‐DTPA, Bayer, Leverkusen, Germany): Due to its strong paramagnetic properties, gadolinium effectively shortens T1 relaxation times and is therefore widely used as a contrast agent in MR imaging, but also to adjust T1 times in test fluids or gels [29].
- Agarose (UltraPure Agarose, Thermo Fisher Scientific, Waltham, MA, USA): Agarose is known to have a strong influence on T2 relaxation times, allowing T2 to be adjusted to tissue‐like values [5, 13].
- Soy lecithin (Carl Roth, Karlsruhe, Germany): Recently, Soy lecithin was identified as a beneficial substance for adjusting the ADC in aqueous solutions to the values of biological tissues: [27, 30] It provides a wide range of adjustable ADC values, shows no additional signal components in the ^1^H spectrum, and has a moderate effect on relaxation times. Being an amphiphilic molecule, soy lecithin is known to form different self‐assembled aggregate structures in aqueous solution, such as uni‐ and multi‐lamellar liposomal vesicles [31, 32, 33], which are assumed to be responsible for the pronounced restriction of water mobility. Using dynamic light scattering (DLS), we measured aqueous soy lecithin solutions at concentrations of 0.05%, 0.1%, 0.25%, and 0.5% wt/vol, which showed mean aggregate sizes in the range of 200 (0.05% wt/vol) to 350 nm (0.5% wt/vol), consistent with vesicle sizes reported by Tsengam et al. [31]

### 2.2 Study Design and Procedure

To design a material with predefined T1, T2, and ADC values, it is essential to understand how the different components—Gd‐DTPA, agarose, and soy lecithin—affect the relaxation and diffusion properties of water, the base solvent. Systematic measurements were performed to determine the relaxivities (r_1_, r_2_) and ADC‐modifying properties of these substances, both individually and in mixtures. To study the MR‐specific properties of substance A (e.g., soy lecithin), samples with increasing concentrations of soy lecithin were prepared, while keeping the other substances (agarose and Gd‐DTPA) constant. These experiments were repeated for different combinations of constant agarose and Gd‐DTPA concentrations to assess whether the diffusion and relaxation properties of soy lecithin are affected by the presence or concentration of the other substances. The same approach was used to determine the relaxation and diffusion‐modifying properties of agarose and Gd‐DTPA. In total, 47 solutions with different concentrations of each substance were prepared, measured, and analyzed. The combinations of Gd‐DTPA, agarose, and soy lecithin ranged from 0 to 0.2 mM (0, 0.05, 0.1, 0.15, 0.2 mM) for Gd‐DTPA, from 0% to 4% (0%, 1%, 2%, 3%, 4% wt/vol) for agarose, and from 0% to 5% (0%, 0.5%, 1%, 2%, 3%, 4%, 5% wt/vol) for soy lecithin. The specific composition of each sample is detailed in Table S1. Based on these preliminary experiments, relationships between MR parameters (T1, T2, and ADC) and the concentrations of the ingredients were derived, and a stepwise preparation procedure was developed.

To validate the preparation protocol, samples were prepared to mimic the relaxation times and ADC values of different tissue types: liver (T1 = 812 ms, T2 = 42 ms, ADC = 1.45 [10^−3^] mm^2^/s), prostate (1597, 80, 1.25 [10^−3^] mm^2^/s), pancreas (725, 43, 0.98 [10^−3^] mm^2^/s), fibroglandular tissue (1444, 54, 1.75 [10^−3^] mm^2^/s), white matter (1084, 69, 0.84 [10^−3^] mm^2^/s) [14, 18, 20, 34, 35, 36, 37, 38, 39]. Note: the liver ADC of 1.45 ⋅ 10^−3^ mm^2^/s represents a strongly perfusion‐influenced value, but was chosen here to provide gels covering a broad range of different values. To evaluate the accuracy (measured versus target values), reproducibility, and temporal stability of the hydrogels, test phantoms were prepared independently three times and examined over a period of 3 months. Batch 1 was measured weekly. Batches 2 and 3 were measured every 2 weeks. To better assess the temporal stability, a pure water sample was also examined as a reference over the same period.

### 2.3 Preparation of the Hydrogels

**FIGURE 1:**
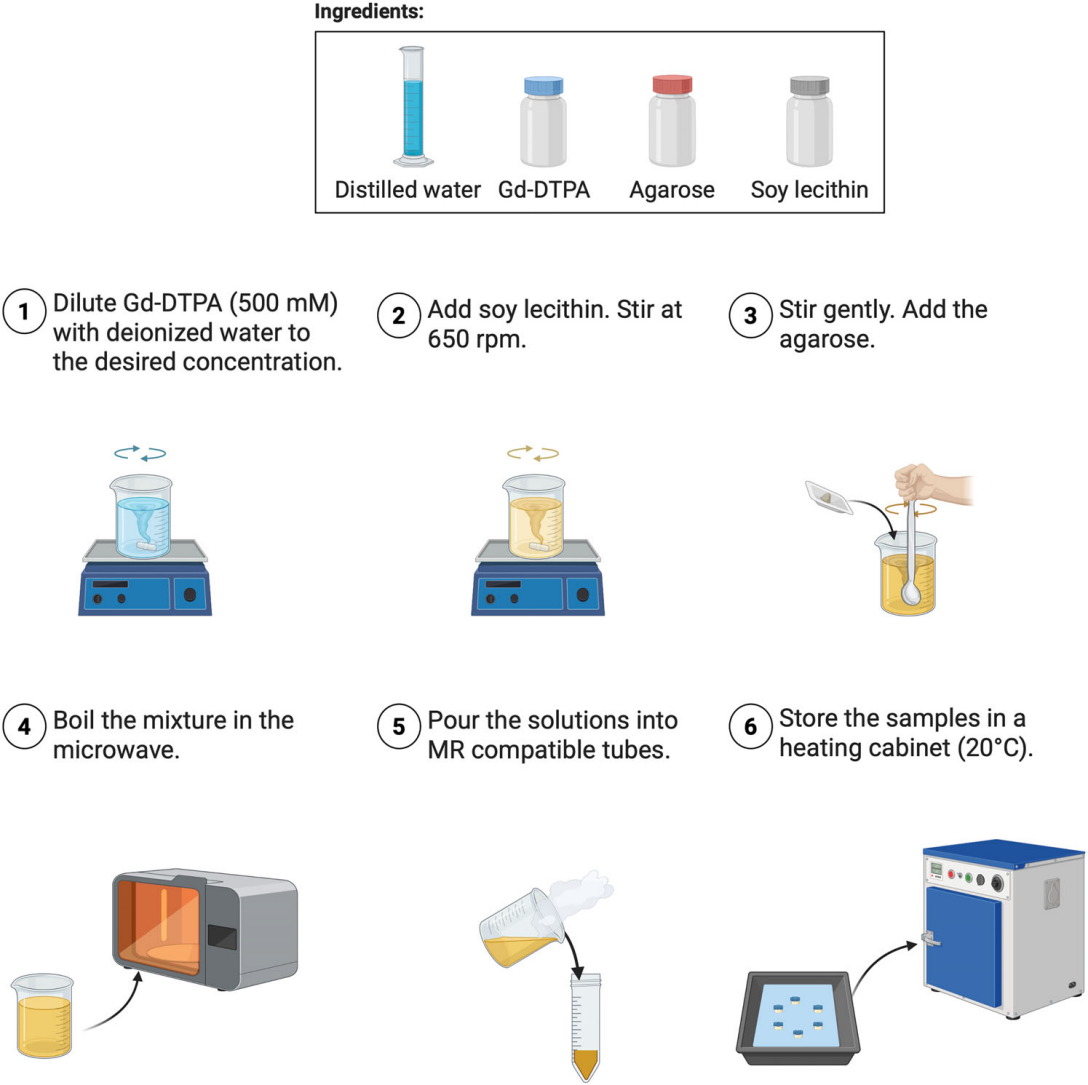
Scheme of the hydrogel preparation process. Created in BioRender. https://BioRender.com/k1h8vah.

Hydrogels were prepared as shown in Figure 1: First, Gd‐DTPA (500 mM) was diluted to the desired concentration with deionized water. Second, soy lecithin was added and dissolved under magnetic stirring at 650 rpm for 10 min. Agarose was then gently stirred into the solution, and the mixture was boiled using a microwave heater until the solution was clear and homogeneous. The solutions were then poured into sterile polypropylene tubes (50 mL, Greiner Bio‐One, Germany) and allowed to cool for gelation. Finally, the prepared hydrogel tubes were placed in water‐filled MR‐compatible housings and stored in a heating cabinet at 20°C.

### 2.4 Data Acquisition and Analysis

Relaxometry and DWI were performed using a clinical 3.0 Tesla scanner (MAGNETOM Prisma^fit^, Siemens Healthcare, Erlangen, Germany) with an 18‐channel body‐array coil. Since the room temperature of the scanner can vary on different measurement days (usually between 19°C and 23°C), the samples were tempered to 20°C as described above. To keep the temperature stable during the measurement period, they were placed in a thermally insulated polystyrene box for the measurement.

T1 relaxation times were measured using a 2D inversion recovery prepared turbo spin echo (IR‐TSE) sequence with nine different inversion times ranging from 25 to 6400 ms. Other sequence parameters were TR = 10,000 ms, TE = 9.9 ms, FOV = 200 × 200 mm, matrix size = 128 × 128, number of slices = 1 (in coronal plane), slice thickness = 5 mm.

T2 measurements were performed in the same slice using a multi‐echo CPMG pulse sequence with 32 echo times ranging from 10 to 320 ms (equally spaced). Imaging parameters were TR = 5000 ms, FOV = 200 × 200 mm, matrix size = 128 × 128, number of slices = 1 (in coronal plane), slice thickness = 5 mm.

DWI was performed using a readout‐segmented echoplanar imaging (RESOLVE‐DWI) sequence with the following parameters: TR = 5000 ms, TE = 48 ms, FOV = 200 × 200 mm, matrix size = 128 × 128, number of slices = 10 (in coronal plane), slice thickness = 5 mm, bandwidth = 751 Hz/px, b‐values = 0, 50, 500, 1000 s/mm^2^, monopolar diffusion sensitizing gradients.

Image processing, including mapping and analysis, was performed offline in MATLAB (R2022b, MathWorks, Natick, MA, USA). T1 and T2 maps were calculated voxel‐wise by mono‐exponential fitting of signal intensities as a function of inversion time (for T1) and as a function of echo time (for T2), respectively. ADC maps were calculated voxel‐wise by a log‐linear fitting of the signal intensities as a function of b‐value. T1, T2, and ADC values of each sample were determined from circular regions of interest in the calculated parametric maps. Relaxivities r_1_ and r_2_ were calculated from the linear regression of the relaxation rates (R1 = 1/T1, R2 = 1/T2) on the concentration of the substance.

## 3 Results

### 3.1 MR Properties of the Ingredients: Gd‐DTPA, Agarose, and Soy Lecithin

**FIGURE 2:**
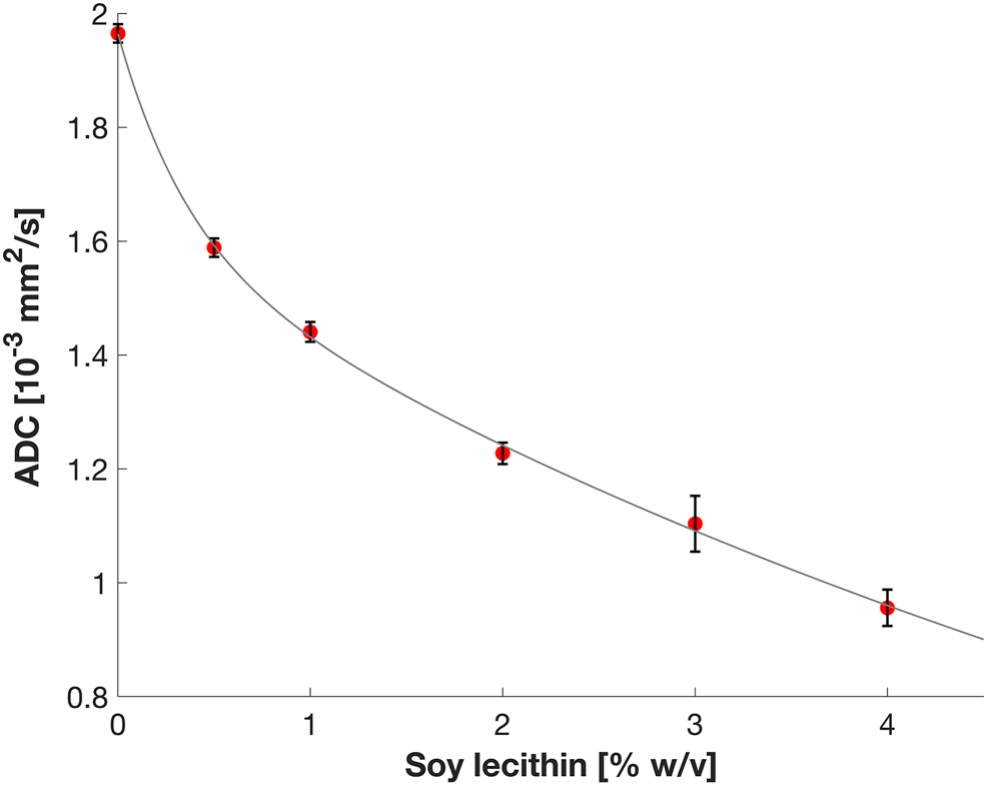
Dependence of ADC on soy lecithin concentration. Each soy lecithin concentration was measured in the presence of different concentrations of agarose (0%, 1%, 2%, 3%, 4% wt/vol) and Gd‐DTPA (0, 0.05, 0.1, 0.15, 0.2 mM). The error bars show the variation in ADC for a given soy lecithin concentration in the presence of different agarose and Gd‐DTPA concentrations.

The dependence of ADC values on soy lecithin concentration was measured under various conditions (in pure water and with different agarose and Gd‐DTPA concentrations). A biexponential relationship was observed between soy lecithin concentration and ADC values, consistent with reported findings for pure aqueous soy lecithin solutions [27]. Our results suggest that the influence of soy lecithin on ADC is almost unaffected by the presence of agarose and Gd‐DTPA (see Figure 2). However, while the general trend remained stable across the conditions tested, variations in the ADC values were observed: At low concentrations of soy lecithin (≤ 2% wt/vol), the standard deviations (SDs) were small, indicating consistent ADC values over repeated measurements under different agarose–Gd‐DTPA compositions. Conversely, at higher concentrations (≥ 3% wt/vol), the SDs were significantly higher, indicating greater variability in ADC values. Unfortunately, this means that the accuracy of the targeted ADC value is lower at higher lecithin concentrations.

For hydrogels with variable amounts of Gd‐DTPA and agarose, the ADC values showed no significant concentration dependence. Therefore, the relationship between ADC and the proposed ingredients is determined only by the soy lecithin concentration with good approximation, as described by the bi‐exponential model in Equation (1):

(1) $$\mathrm{ADC}\;\left[10^{-3}\right]=0.36\cdot e^{-2.79\cdot C_{\mathrm{lec}}\;}+1.60\cdot e^{-0.13\cdot C_{\mathrm{lec}}\;}$$

Relaxivities (r_1_, r_2_) for soy lecithin, Gd‐DTPA, and agarose were determined for pure aqueous solutions with only one ingredient and for mixtures at varying concentrations of the other components (results are summarized in Table 1). For soy lecithin and Gd‐DTPA, the relaxivities were r_1,lec_ = 0.10 s^−1^ (% wt/vol)^−1^, r_2,lec_ = 0.69 s^−1^ (% wt/vol)^−1^, r_1,Gd_ = 3.78 s^−1^ mM^−1^, and r_2,Gd_ = 4.24 s^−1^ mM^−1^. These values closely match their individual relaxivities (shown in parentheses in Table 1), suggesting that their effects on the relaxation times of water remain unchanged in the presence of the other components. For agarose, however, this is only true for T2. The determined transverse relaxivity of agarose is r_2,agarose_ = 6.23 s^−1^ (% wt/vol)^−1^, consistent with its individual transverse relaxivity. In contrast, the longitudinal relaxivity r_1,agarose_ could not be reliably determined in the presence of Gd‐DTPA and soy lecithin, since no significant dependence of the relaxation rate R1 (1/T1) on the agarose concentration was observed. This means that the contribution of agarose to T1 is negligible in the composite hydrogel. All three ingredients—Gd‐DTPA, soy lecithin, and agarose—affect the transverse relaxation time T2, while the longitudinal relaxation time T1 is solely dependent on Gd‐DTPA and soy lecithin concentrations. Consequently, the relaxation properties of the composite hydrogel can be mathematically modeled as presented in Equations (2) and (3):

(2) $$R1=R1_{w}+r_{1,\mathrm{Gd}}\cdot \;C_{\mathrm{Gd}}+r_{1,\mathrm{lec}}\cdot \;C_{\mathrm{lec}}$$

(3) $$R2=R2_{w}+r_{2,\mathrm{Gd}}\cdot \;C_{\mathrm{Gd}}+r_{2,\mathrm{lec}}\cdot \;C_{\mathrm{lec}}+r_{2,a}\cdot \;C_{a}$$

where R1 and R2 are the relaxation rates (1/T_1,2_) of the gels, R1_w_ and R2_w_ are the relaxation rate of pure water, C_Gd_ is the concentration of Gd‐DTPA in mM, C_lec_, and C_a_ are the concentrations of soy lecithin and agarose in % (wt/vol), and r_1_ and r_2_ are the longitudinal‐ and transverse relaxivities of each substance.

**TABLE 1:** Relaxivities r1 and r2 of Gd‐DTPA, agarose, and soy lecithin.

	Gd‐DTPA (mM^−1^ s^−1^)	Agarose (s^−1^ [% wt/vol]^−1^)	Soy lecithin (s^−1^ [% wt/vol]^−1^)
r_1_	3.78 ± 0.14 (3.62)	N/A (0.02)	0.10 ± 0.02 (0.12)
r_2_	4.24 ± 0.16 (4.33)	6.62 ± 0.47 (5.65)	0.69 ± 0.04 (0.70)

### 3.2 Gel Preparation Protocol

Since the ADC value depends only on the soy lecithin concentration, the T1 value on the concentrations of soy lecithin and Gd‐DTPA, and the T2 value on the concentrations of soy lecithin, Gd‐DTPA, and agarose, the determination of the component concentrations follows a three‐step procedure: (1) the soy lecithin concentration is determined based on the target ADC value using Equation (1) to achieve the desired diffusion characteristics, (2) with the soy lecithin concentration determined, the Gd‐DTPA concentration is calculated to achieve the target T1 relaxation time using Equation (2), and (3) finally, the agarose concentration is determined using the calculated soy lecithin and Gd‐DTPA concentrations along with the target T2 relaxation time using Equation (3).

### 3.3 Achievable Combinations of T1, T2, and ADC

**FIGURE 3:**
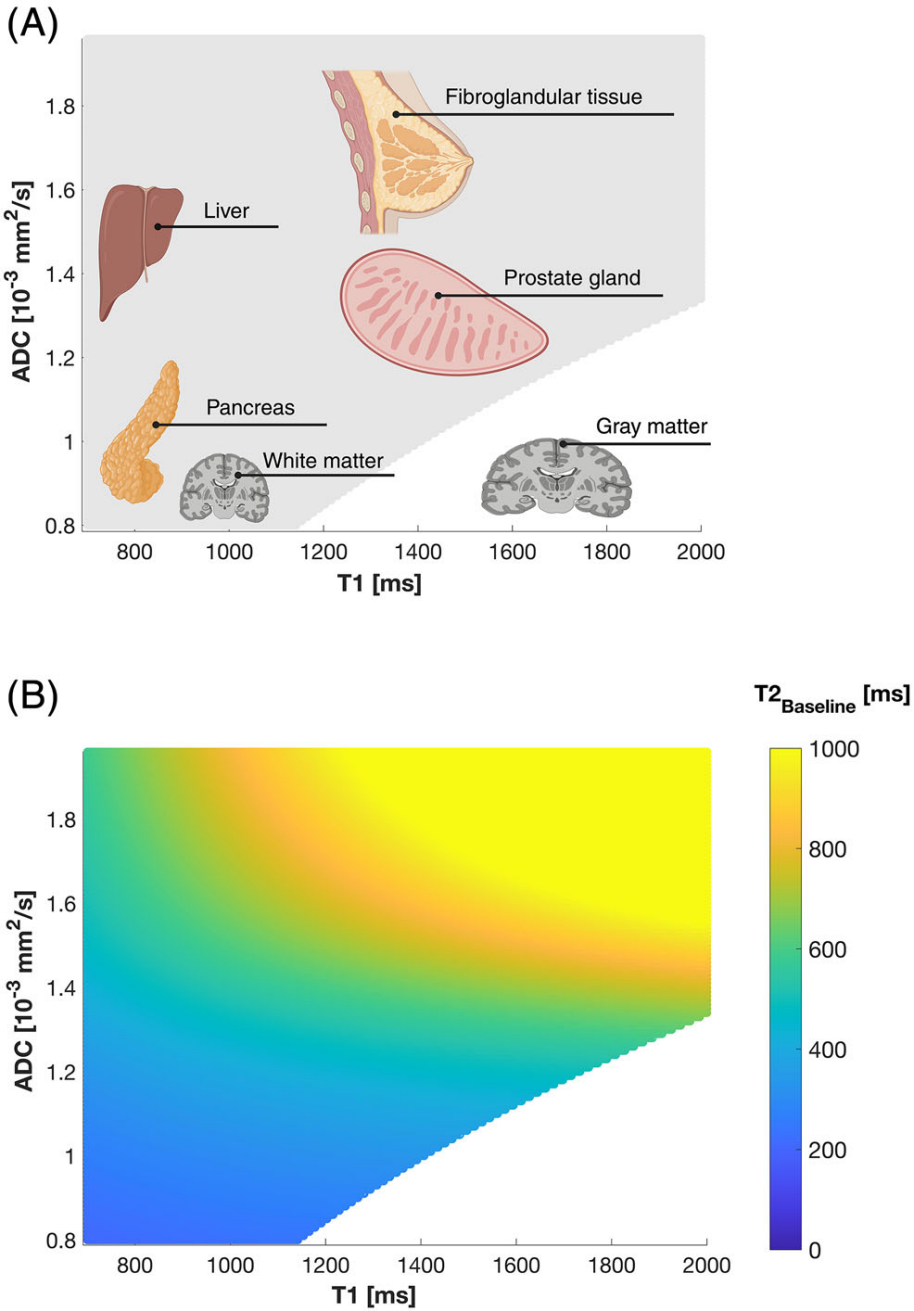
(A) Achievable combinations of ADC and T1 (gray area) using the proposed recipe. Achievable values cover most human tissues such as liver, prostate, and white matter. Only tissues with low ADC values but long T1 times (e.g., gray matter) are not included in the accessible range. (B) Baseline T2 values as a function of selected ADC and T1 values before addition of agarose. For all combinations of ADC and T1 considered, the baseline values remained above 200 ms, providing sufficient flexibility for further adjustment to physiologically relevant T2 values by adding agarose. Created in BioRender. https://BioRender.com/yu9nc7d.

Due to the described secondary effects of the ingredients, not all combinations of T1, T2, and ADC can be realized with the proposed approach. Soy lecithin, which is used to adjust the ADC in step (1), also reduces T1 to a non‐negligible extent (r_1,lec_ = 0.10 s^−1^ (% wt/vol)^−1^). As a result, the achievable T1 values are highly dependent on the selected ADC: The lower the ADC value selected, the more limited is the range of achievable T1 times (Figure 3A). For example, if ADC is set to 0.9 mm^2^/s, only T1 values ≤ 1400 ms can be reached. Nevertheless, the range of combinations (T1, T2, ADC) is very wide and includes most types of tissue in the human body, for example, liver, prostate, and white matter [14, 18, 20, 35, 37, 38, 39]. Only a few tissues, such as gray matter (ADC = 0.8–1.0 [10^−3^] mm^2^/s, T1 = 1500–1850 ms) [18, 34, 36], are outside the range and cannot be well simulated.

The adjustable T2 range depends on the preselected ADC and T1. Due to the T2‐shortening effects of soy lecithin and Gd‐DTPA—used in the preceding steps to set ADC and T1—the baseline T2 is already reduced to some extent without the addition of agarose. Figure 3B illustrates the baseline T2 times as a function of the selected ADC and T1. The ADC range in Figure 3 includes values from 0.8 to 2 [10^−3^] mm^2^/s while T1 from 700 to 2000 ms are considered. These ranges cover properties of almost all tissue types. For all (T1, ADC) combinations available, the baseline T2 remained above 200 ms. This provides relatively high flexibility for further T2 adjustments by the addition of agarose, as T2 in most biological tissues is smaller than 100 ms [18].

### 3.4 Experimental Validation of the Preparation Protocol

**FIGURE 4:**
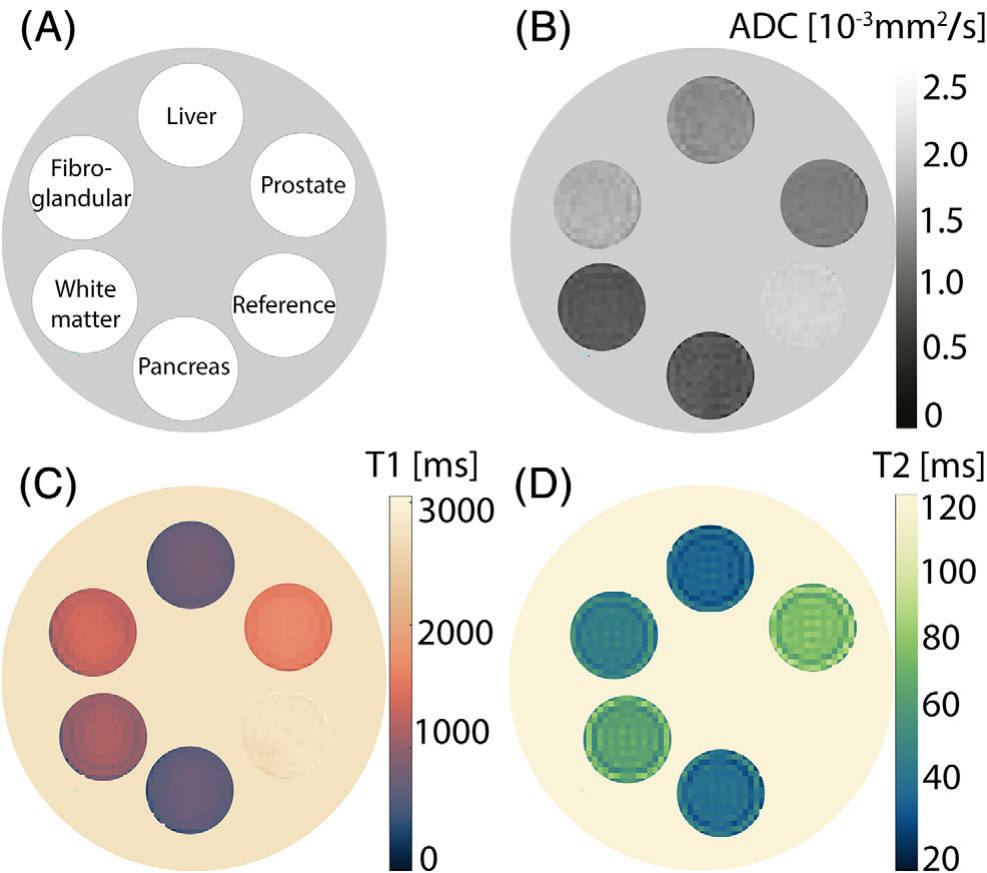
Parametric maps of gels in the test phantoms mimicking relaxation times of different tissues (pancreas, white matter, fibroglandular tissue, liver, and prostate). (A) Representation of the positions of the hydrogel‐filled tubes in the cylindrical housing. (B) Parametric map of ADC values. (C) Parametric map of T1 times. (D) Parametric map of T2 times. The color maps were selected according to the recommendations of the MR Quantitative Study Group of the International Society of Magnetic Resonance in Medicine (ISMRM) [40, 41].

Test phantoms with hydrogels mimicking the diffusion and relaxation properties of different tissues (pancreas, white matter, fibroglandular tissue, liver, and prostate) were prepared according to the presented protocol. The composition of the phantoms (three identical phantoms were prepared in order to test variability) is shown in Table S2. The hydrogels were then tested for accuracy (in terms of deviations from targeted values), reproducibility, and temporal stability. The targeted and measured values for ADC, T1, and T2 are listed in Table 2, presented as means and standard deviations across the three batches. The corresponding parametric maps are shown in Figure 4A–D as an example for Batch 1. All hydrogels provided a reasonable agreement between measured and intended values: Deviations were less than 8% for T1, less than 7.5% for T2, and less than 11.5% for ADC. Excluding the pancreas mimicking gel, the deviations were even less than 3% for ADC. In addition, the preparation of the gels showed a reasonable reproducibility, as evidenced by the relatively low standard deviations observed across the three batches (Table 2), underscoring the robustness and accuracy of the preparation protocol.

**TABLE 2:** Target and measured values for T1, T2, and ADC, presented as mean and standard deviation across the three batches.

	T1 (ms)		T2 (ms)		ADC (10^−3^ mm^2^/s)	
	Target	Measured	Target	Measured	Target	Measured
Pancreas	725	757 ± 16	43	43 ± 1	0.98	0.87 ± 0.01
White matter	1084	1143 ± 3	69	66 ± 1	0.84	0.83 ± 0.04
Fibroglandular	1444	1396 ± 13	54	50 ± 1	1.75	1.75 ± 0.02
Liver	812	809 ± 14	42	41 ± 1	1.40	1.43 ± 0.03
Prostate	1597	1721 ± 28	80	80 ± 2	1.25	1.23 ± 0.02

**FIGURE 5:**
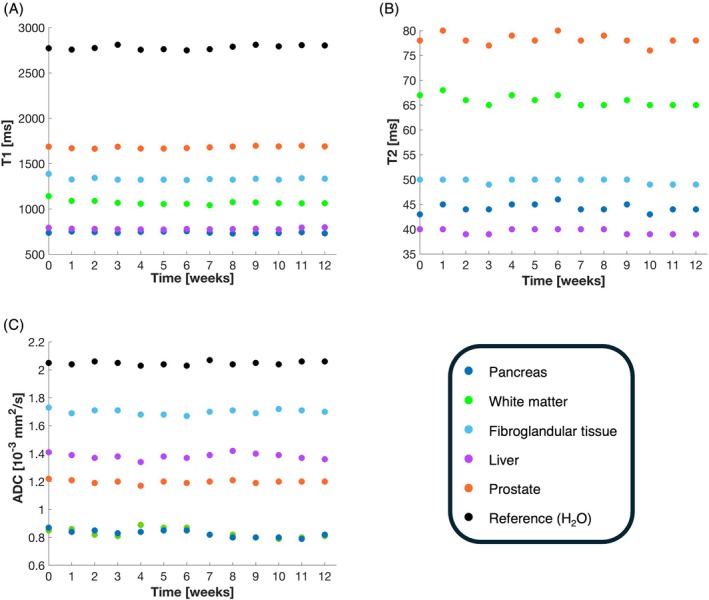
Temporal stability of the prepared hydrogels (Batch 1) with respect to T1 (A), T2 (B), and ADC (C).

Temporal stability was also acceptable: Over a 12‐week storage period, the ADC values and relaxation times of the hydrogel‐filled tubes in the phantoms did not change significantly (Figures 5, S1, S2). Small variations were observed between the measurements, but these were also present in the reference sample (pure water) and are therefore expected to be due to measurement uncertainties.

## 4 Discussion

To date, no instructions have been described for test materials that allow simultaneous and independent adjustment of relaxation and diffusion properties [1]. Most existing materials for test phantoms are designed to mimic only one or two of these parameters, while the other(s) are not controlled. This work presented a practical and reproducible method for the preparation of hydrogels with adjustable T1, T2, and ADC values over a wide range so that many tissue types can be well simulated. The gels are easy to prepare, inexpensive, and show an acceptable temporal stability of at least 3 months. The deviations between measured and target values were less than 8% for relaxation times (T1, T2) and even less than 3% for ADC (except for the pancreas‐mimicking gel), confirming the robustness and reliability of the preparation protocol. The comparatively higher deviation observed in the pancreas‐mimicking gel (11.5% for ADC) can likely be attributed to the higher variability in ADC measurements at high soy lecithin concentrations. It was shown that at soy lecithin concentrations above 3% (wt/vol), ADC values exhibited a slight dependence on agarose‐Gd‐DTPA compositions (see *Results, MR Properties of the Ingredients: Gd‐DTPA, Agarose, and Soy Lecithin*). This could be due to subtle interactions between soy lecithin and the other substances (agarose and Gd‐DTPA), which become more pronounced at higher soy lecithin concentrations and are not accounted for in the parameter adjustment equations. This interpretation is further supported by the high reproducibility of the pancreas‐mimicking gel: although the deviation in ADC was larger compared to the other phantom gels, it was consistently observed across repeated preparations (three batches). This suggests that the effect is more likely related to systematic interactions between soy lecithin, agarose, and Gd‐DTPA, rather than to reduced reliability of the preparation at higher lecithin concentrations.

Although the hydrogels were successfully produced and allowed for simultaneous and independent adjustment of T1, T2, and ADC values, some limitations remain and must be considered. The presented work focused on developing a formulation that enables relaxation times and ADC to be adjusted largely independently. The range considered includes ADC values from 0.8 to 2 [10^−3^] mm^2^/s, T1 values from 700 to 2000 ms, and T2 values from 100 to 40 ms. While this covers a broad spectrum of clinically relevant tissue properties, full independence of parameters could not be achieved across the entire range. In particular, the interplay between ADC and T1 proved challenging, since soy lecithin affects both parameters simultaneously. As a result, tissue types characterized by relatively low ADC but long T1 values, such as gray matter, could not be accurately mimicked with the proposed formulation. Furthermore, for certain oncological applications, such as prostate cancer staging, even lower ADC thresholds (<0.8 ⋅ 10^−3^ mm^2^/s) are of interest [42, 43]. In principle, further reduction is possible by increasing the soy lecithin concentration (above 5% wt/vol). A previous study has shown that the addition of soy lecithin can reduce water diffusion to values below 0.5 ⋅ 10^−3^ mm^2^/s at room temperature [27]. This, however, comes with markedly shortened T1 times, which may compromise physiological realism. Additionally, high agarose concentration (3%–4% wt/vol) required for relatively short T2 times becomes challenging when combined with these high soy lecithin concentrations (> 5% wt/vol). This combination leads to a significant increase in gel viscosity, which makes homogeneous mixing difficult and increases the incorporation of air bubbles [25]. This is also the reason why the present study focused on T2 times above 40 ms, which still cover many clinically relevant tissues [18]—shorter T2 times would require even higher agarose concentrations and, as described, become critical for phantom preparation.

The presented design (Equations (1), (2), (3)) does not account for temperature‐ and field dependence. In this study, all measurements were performed at a temperature of 20°C and a field strength of 3 T. However, it is well known that diffusion and relaxation properties are temperature dependent and that relaxation times are also influenced by magnetic field strength [44, 45, 46, 47]. These dependencies may lead to discrepancies in measurements under different scanning conditions. Therefore, further measurements at different temperatures and field strengths are needed to systematically study the effects of these factors and to refine the preparation protocol to make it more practical. In addition, research is needed to evaluate potential long‐term drifts in relaxation and diffusion properties. Factors such as dehydration or structural changes in agarose or soy lecithin could affect stability over time. Long‐term stability (> 12 weeks) is essential when phantoms are used in reproducibility or multicenter studies where measurements are taken over several months or even years. However, for the intended use in research to develop and validate prototype MRI sequences and provide proof of concept, the stability of the hydrogels appears to be sufficient for most applications. Furthermore, the presented gels are designed to mimic specific relaxation and diffusion properties but do not take into account other tissue properties that can significantly affect the MR signal. Effects such as magnetization transfer, non‐Gaussian diffusion (kurtosis), susceptibility, or perfusion‐related signal contributions are not controlled or specifically adjustable within the current test phantom designs. Future developments could aim to integrate or approximate additional tissue‐related parameters to further improve physiological realism. Measurements with higher b‐values of up to 3000 s/mm^2^ could enable a more detailed characterization of the diffusion behavior of the gels, including kurtosis. It is also important to note that Equations ((1), (2), (3)) in the preparation protocol apply only to the product sources used in this study. Soy lecithin and agarose are natural products, so there may be differences in composition or purity from product to product, and thus in MR properties. The authors recommend that when using products other than those presented here, the components should first be characterized by determining their relaxivities and ADC‐modifying properties, and constant factors in Equations ((1), (2), (3)) should be adapted appropriately.

Another important aspect, which was beyond the scope of the present study, is a systematic characterization of the morphology and size distribution of the aggregates formed by soy lecithin to better understand the observed restriction of water mobility and MR signal behavior. Preliminary DLS measurements of aqueous soy lecithin solutions indicated mean aggregate sizes ranging from ˜200 nm at a concentration of 0.05% wt/vol to 350 nm at a concentration of 0.5% wt/vol. The observed increase in size with concentration suggests that the aggregates do not simply increase in number but may also undergo changes in morphology and size [32, 33]. This, in turn, supports the finding that the relationship between ADC and soy lecithin concentration is not linear. Future studies should investigate the underlying physicochemical structures in more detail. Additional experiments with small‐angle x‐ray scattering (SAXS) and cryogenic transmission electron microscopy (cryo‐TEM) could provide deeper insights into the underlying mechanisms [31, 48, 49], also at higher concentrations of up to 5% wt/vol.

In summary, the paper presents a step‐by‐step protocol for the preparation of hydrogels with independently adjustable T1, T2, and ADC values at 3 T using soy lecithin, agarose, and Gd‐DTPA. The preparation process is very straightforward, reproducible, and relatively cost‐effective. Furthermore, the gels have an acceptable temporal stability of at least 3 months, making them suitable as reference materials for MRI research. Nevertheless, further work is needed to investigate temperature‐ and field dependency as well as the long‐term stability (> 12 weeks) of the materials. To facilitate the practical use of the proposed recipe, we provide a Python‐based calculator that determines the required concentrations of Gd‐DTPA, agarose, and soy lecithin for user‐defined T1, T2, and ADC target values for other users on request (Figure S3).

## Supporting information


**Figure S1:** Temporal stability of the hydrogels in Batch 2 with respect to T1 (A), T2 (B), and ADC (C).
**Figure S2:** Temporal stability of the hydrogels in Batch 3 with respect to T1 (A), T2 (B), and ADC (C).
**Figure S3:** Python‐based calculator for the determination of the required concentrations of Gd‐DTPA, agarose, and soy lecithin for user‐defined T1, T2, and ADC target values.
**Table S1:** Composition of solutions with different concentrations of soy lecithin, Gd‐DTPA, and agarose. A total of 46 solutions were prepared, measured, and analyzed to determine relaxation and diffusion‐modifying properties of the ingredients for the hydrogels.
**Table S2:** Composition of hydrogels mimicking the diffusion‐ and relaxation properties of different tissues.
