# Supplementary material for: Recipe for Hydrogels With Tunable Relaxation and Diffusion Properties for Use as MRI Test Materials
Source: Magn Reson Med. 2025 Oct 5;95(3):1823–32. doi: 10.1002/mrm.70120 (PMC12746408; doi:10.1002/mrm.70120)
Supplement: Supplementary file 1 — Figure S1: Temporal stability of the hydrogels in Batch 2 with respect to T1 (A), T2 (B), and ADC (C). Figure S2: Temporal stability of the hydrogels in Batch 3 with respect to T1 (A), T2 (B), and ADC (C). Figure S3: Python‐based calculator for the determination of the required concentrations of Gd‐DTPA, agarose, and soy lecithin for user‐defined T1, T2, and ADC target values. Table S1: Composition of solutions with different concentrations of soy lecithin, Gd‐DTPA, and agarose. A total of 46 solutions were prepared, measured, and analyzed to determine relaxation and diffusion‐modifying properties of the ingredients for the hydrogels. Table S2: Composition of hydrogels mimicking the diffusion‐ and relaxation properties of different tissues. [file MRM-95-1823-s001.pdf]

Supporting Information

Figures

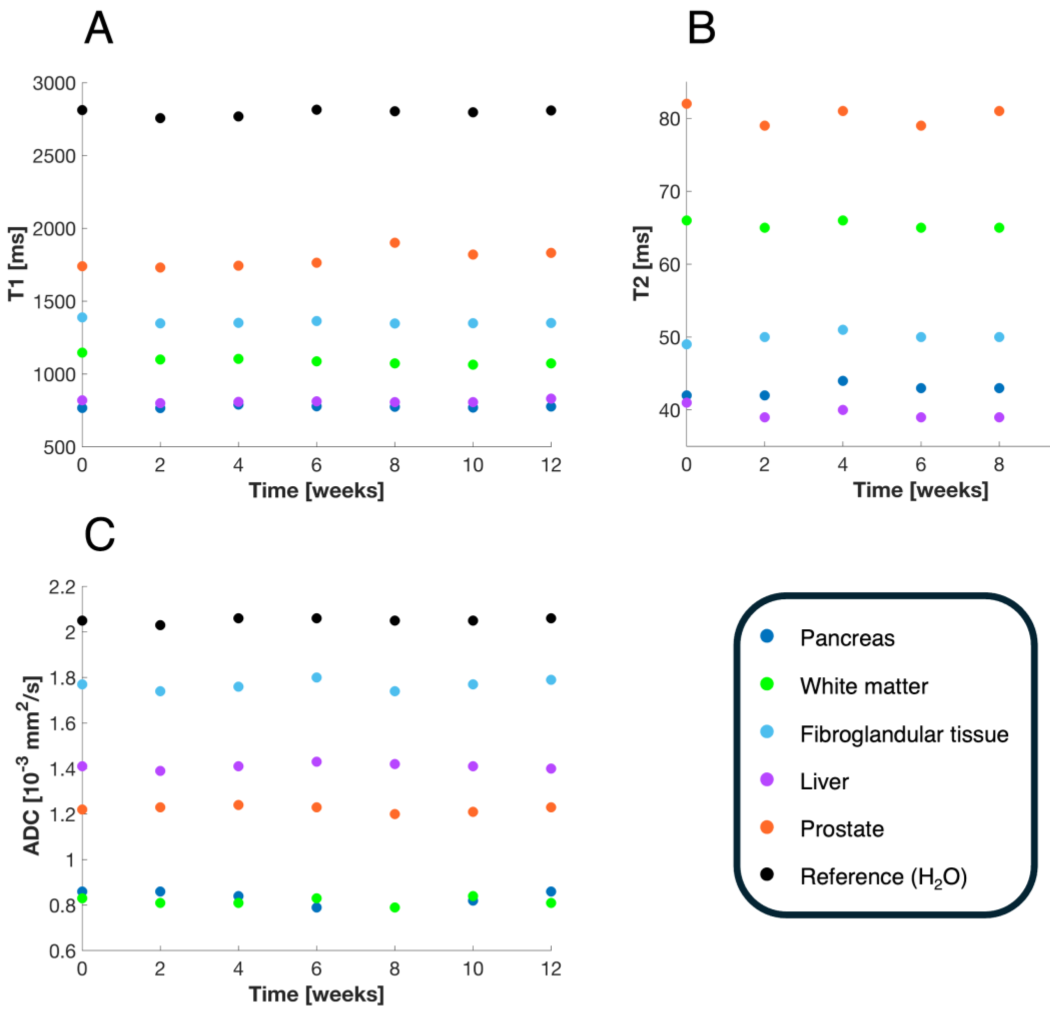

Figure S1. Temporal stability of the hydrogels in Batch 2 with respect to T1 (A), T2 (B) and ADC (C).

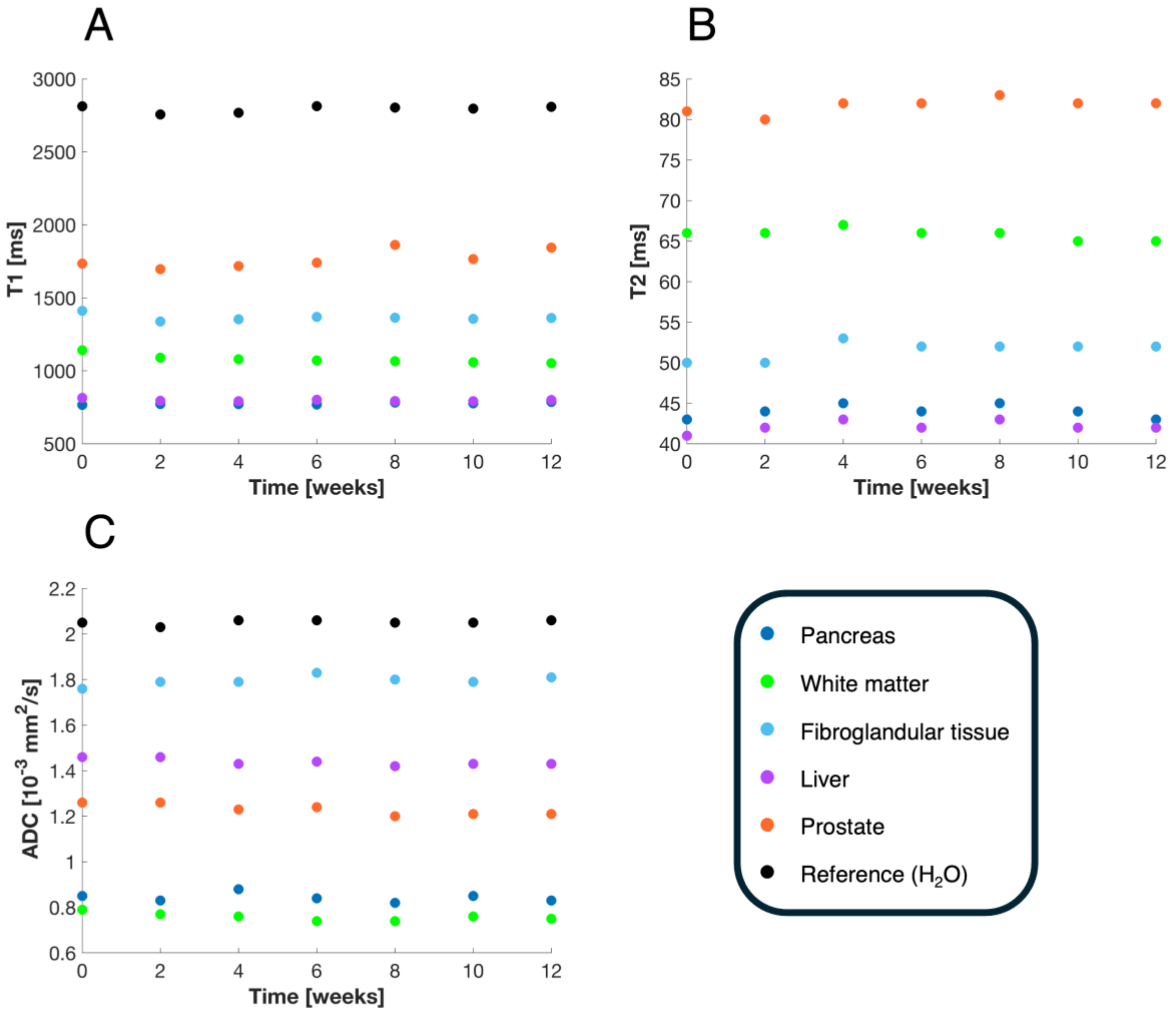

Figure S2. Temporal stability of the hydrogels in Batch 3 with respect to T1 (A), T2 (B) and ADC (C).

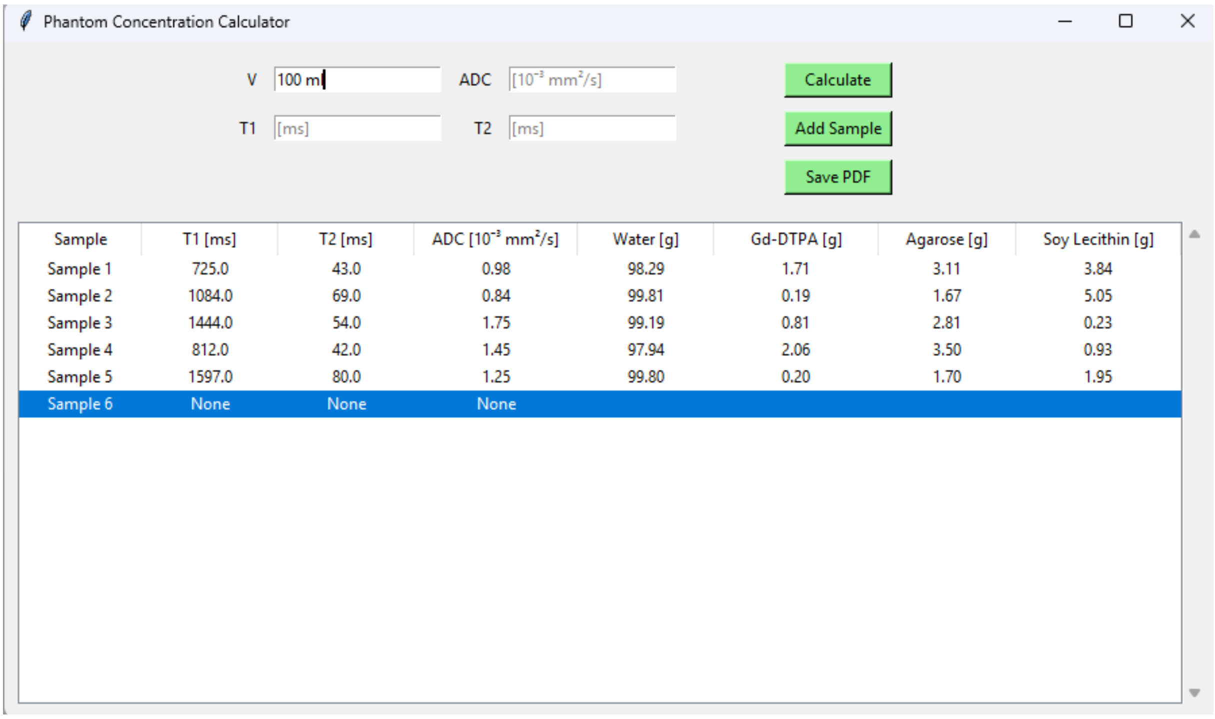

Figure S3. Python-based calculator for determination of the required concentrations of Gd-DTPA, agarose, and soy lecithin for user-defined T1, T2, and ADC target values.

Tables

	Soy lecithin [% w/v]	Gd-DTPA [mM]	Agarose [% w/v]
Sample 1	0	0	0
Sample 2	0.5	0	0
Sample 3	1	0	0
Sample 4	2	0	0
Sample 5	3	0	0
Sample 6	4	0	0
Sample 7	5	0	0
Sample 8	0	0.05	0
Sample 9	0	0.10	0
Sample 10	0	0.15	0
Sample 11	0	0.02	0
Sample 12	0	0	1
Sample 13	0	0	2
Sample 14	0	0	3
Sample 15	0	0	4
Sample 16	0.5	0.05	1
Sample 17	0.5	0.05	3
Sample 18	0.5	0.15	1
Sample 19	0.5	0.15	3
Sample 20	1	0.05	1
Sample 21	1	0.05	3
Sample 22	1	0.15	1
Sample 23	1	0.15	3
Sample 24	2	0.05	1
Sample 25	2	0.05	3
Sample 26	2	0.15	1
Sample 27	2	0.15	3
Sample 28	3	0.05	1
Sample 29	3	0.05	3

Sample 30	3	0.15	1
Sample 31	3	0.15	3
Sample 32	4	0.05	1
Sample 33	4	0.05	3
Sample 34	4	0.15	1
Sample 35	4	0.15	3
Sample 36	1	0.10	1
Sample 37	1	0.10	3
Sample 38	4	0.10	1
Sample 39	4	0.10	3
Sample 40	1	0.05	1.5
Sample 41	1	0.15	1.5
Sample 42	4	0.05	1.5
Sample 43	4	0.15	1.5
Sample 44	1	0.05	2.5
Sample 45	1	0.15	2.5
Sample 46	4	0.05	2.5
Sample 47	4	0.15	2.5

Table S1: Composition of solutions with different concentrations of soy lecithin, Gd-DTPA and agarose. A total of 46 solutions were prepared, measured and analyzed to determine relaxation and diffusion modifying properties of the ingredients for the hydrogels.

	Soy lecithin [% w/v]	Gd-DTPA [mM]	Agarose [% w/v]
Pancreas	3.84	0.171	3.11
White matter	5.05	0.019	1.67
Fibroglandular	0.23	0.081	2.81
Liver	0.93	0.206	3.50
Prostate	1.95	0.020	1.70

Table S2: Composition of hydrogels mimicking the diffusion- and relaxation properties of different tissues.
